# Behavioural, demographic and fitness consequences of social instability in cooperatively breeding dwarf mongoose groups

**DOI:** 10.1098/rspb.2023.0901

**Published:** 2023-08-30

**Authors:** Julie M. Kern, Amy Morris-Drake, Andrew N. Radford

**Affiliations:** ^1^ School of Biological Sciences, University of Bristol, 24 Tyndall Avenue, Bristol BS8 1TQ, UK; ^2^ School of Environmental and Rural Science, University of New England, Armidale 2351, NSW, Australia

**Keywords:** animal behaviour, social instability, cooperation, cooperative breeding

## Abstract

Social instability frequently arises in group-living species, but the potential costs have rarely been investigated in free-living cooperative breeders, especially across different timeframes. Using natural observations, body mass measurements and life-history data from dwarf mongooses (*Helogale parvula*), we determined the short- and long-term consequences of a change in one of the dominant breeding pairs. We found that a new breeder led to alterations in both collective and individual behaviours (i.e. increases in communal scent-marking, engagement in intergroup interactions, sentinel activity and within-group grooming), as well as reduced body mass gain, further demographic changes and decreased reproductive success (i.e. fewer pups surviving to adulthood). The effects were particularly apparent when it was the female breeder who changed; new female breeders were younger than more experienced counterparts. Our findings support the idea that stability and cooperation are strongly linked and provide potential reasons for previously documented health and fitness benefits of social stability.

## 1. Introduction

In social species, there are considerable health and fitness benefits for individuals in groups with stable membership and relationships [1–3]. To understand the reasons for these benefits, many studies have assessed how they increase with greater social stability. For example, the development of stronger bonds between individuals has been shown to improve longevity, offspring survival and reproductive success [2,3], while the strengthening of networks among group members can also enhance survival [4,5]. However, insight can also be obtained by investigating the *costs* of social *instability* [6,7].

Social instability can arise through changes in group membership, the rank order of individuals in a group's hierarchy and/or a reduction in the orderliness of the intragroup aggression network [7]. Changes in adult group composition (membership instability)—resulting from deaths, immigration and emigration—likely generate heightened stress [8–10]. This stress, or alterations in group size and inter-individual relationships caused by the loss or gain of group members, can affect behaviour and generate individual-level costs. Previous studies have mostly investigated short-term impacts, reporting reductions in foraging efficiency [6,11], allogrooming [8,11], food sharing [12,13] and alloparental care [14], as well as increases in aggression [15,16]. Beyond short-term behavioural changes, membership instability might also generate long-term consequences such as further demographic turnover [17,18], reduced reproductive success [14,19,20] and group extinction [21], but rarely have the potential costs of instability across different timeframes been investigated in the same species. Moreover, despite social stability being considered a key factor in the evolution of cooperative societies [22,23], the costs of instability have received limited attention in cooperative breeders and, to the best of our knowledge, have never been considered across multiple timescales.

In cooperatively breeding species, either just one dominant pair or a small subset of group members reproduce; non-breeding, subordinate ‘helpers’ assist in rearing the offspring [24]. This breeding system is found in, for example, approximately 9% of birds and approximately 2% of mammal species [25,26]. The loss of any individual from a cooperatively breeding group may have consequences for those remaining: for instance, while individuals could benefit from reduced foraging or mating competition, they might also have to increase their helping effort to compensate for a smaller group size [27,28]. However, given their disproportionate influence on group decision-making [29], and that social instability is expected to be more impactful if it occurs in the top part of a hierarchy [30,31], it is the loss of breeders that is likely especially disruptive. Much is known about the aggressive power struggles among remaining group members and/or outsiders when there is a breeding vacancy [15,16,32], but less attention has been paid to behavioural, demographic and reproductive changes following breeder replacement. Moreover, while the magnitude of the effects might be expected to differ depending on breeder sex—there is some evidence from grey wolves (*Canis lupus*) that it is more destabilizing to lose the female compared to the male breeder [21]—this has received little formal testing.

Here, we use long-term data from a wild population of cooperatively breeding dwarf mongooses (*Helogale parvula*) to investigate the consequences of membership instability arising from a change in breeder. Dwarf mongoose groups comprise a dominant breeding pair and non-breeding subordinate helpers of both sexes (mean ± s.e. adult group size = 8.0 ± 0.2, range = 3–17; *N* = 62 group-years of study). All adult group members perform a range of cooperative behaviours besides offspring care, including allogrooming (hereafter grooming), territory defence and acting as a sentinel [33,34]. Wild dwarf mongooses can be habituated to the close presence of observers, allowing the collection of detailed, ecologically valid data on behaviour, body mass and life-history events in multiple groups [35,36]. We predicted that a breeder change, especially of the female member of the dominant pair, would lead to changes in collective and individual behaviour (e.g. increased territorial defence, decreased contributions to cooperative acts), reduced body mass gains, further demographic changes (e.g. outgroup members immigrating or additional breeder changes) and a decrease in reproductive success.

## 2. Materials and methods

### (a) Study site and species

Data were collected at Sorabi Rock Lodge, Limpopo Province, South Africa (24° 11′ S, 30° 46′ E), the site of the long-term Dwarf Mongoose Research Project (DMRP). Study groups are habituated to close (less than 5 m) human presence [35] and were monitored for varying periods between July 2011 and May 2021 (mean ± s.e. days per group = 2025 ± 336, range = 714–3627). Adult group members (greater than 12 months old) are classified as either breeders (male and female dominant pair) or subordinate helpers. The dominant pair is readily and unambiguously identified through observations of aggression, feeding displacement, scent-marking and reproductive behaviour [28,35,37]. Individuals are sexed through observations of ano-genital grooming [35,38]. Each study group is generally visited for 2–3 days a week to maintain habituation, to reapply small marks of blonde hair-dye (Wella UK Ltd, Surrey, UK) used for individual identification, and to collect behavioural, body mass and life-history data. The DMRP comprises four field-team members throughout the year; new team members are rigorously trained by a Field Manager before collecting data alone, with all data entry carefully checked by both the Field Manager and a Data Manager in the UK. Team members are rotated across all available groups to ensure equal effort. Observation sessions coincide with the emergence of dwarf mongoose groups from underground burrows. During summer months, two sessions (3–4 h in the morning and in the afternoon) are conducted daily (mongooses retreat below ground during the hottest hours), but full-day sessions are conducted during winter; only rarely (e.g. in thunderstorms) are observation sessions cut short. Behavioural observations and body mass data were analysed from paired two-week periods either side of a breeder change (see below); paired periods were at the same time of year (summer or winter) thus with the same observation-session regime. Work was conducted under permission from the Limpopo Department of Economic Development, Environment and Tourism (permit number: 001-CPM403-00013), and the ethical approval committees of the University of Bristol, UK and Pretoria University, South Africa.

### (b) Data collection

During each observation session, data on group movement and territorial behaviour were collected. Once groups left the burrow to begin foraging, continuous movement data were collected using a GPS device (Garmin, Kansas, USA) held by the observer which recorded track position every 10 s. Data were imported into Mapsource (software version 6.16.3, Garmin Ltd) and each observation-session's track stored as a movement map [33]. Territorial behaviour takes two main forms in dwarf mongooses: infrequent intergroup interactions (IGIs), in which two neighbouring groups physically encounter each other [37], and regular latrining behaviour, where group members deposit scent-marks at communal latrine sites [33,39]. All observed IGIs (when two groups were in visual or acoustic contact) and latrine events (when group members interacted with a known latrine by sniffing and/or depositing scent) were noted.

During each observation session, data on individual contributions to intragroup affiliation (grooming) and sentinel activity (raised guarding) were also collected. All observed grooming, which primarily takes place at overnight sleeping refuges, was recorded; the identity of grooming partners in all bouts of greater than 5 s was noted, with bouts considered finished if 10 s elapsed without any grooming [34]. Sentinel scan samples were carried out every 30 min during daily foraging, recording whether a sentinel was present and, if so, the sentinel's identity [28]. Sentinels were defined as individuals positioned on an object (e.g. termite mound, tree), with their hind feet at least 10 cm above the surrounding substrate, and actively scanning the surroundings while groupmates were engaged in other activities, primarily foraging [28,34].

The study population is habituated to the use of weighing scales, and most individuals will stand on an electronic balance (Salter Houseware, Kent, UK, accuracy ± 1 g) for a small reward of egg. During morning sessions, individuals were weighed after emergence from their overnight refuge and again after a 3 h foraging session, to determine body mass changes.

During each observation session, the full group composition was recorded, enabling collection of life-history data (e.g. breeder changes, immigration, emigration, births and deaths). As the dominant pair can be readily identified (see above), it is promptly apparent when there is a breeder change. For every breeder change, breeder sex, whether the change arose from an active displacement or a vacancy, and whether the new breeder was an existing group member (internal) or an outsider (external) was noted. Displacements were defined as the supplanting of an existing breeder by a rival and vacancies as the opening of a breeding position following the death/dispersal of the outgoing breeder. Individuals were classified as internal when they had been a subordinate group member for at least 30 days (even if they had previously joined the group as an immigrant), and as external when they were an outsider who joined directly as a new breeder. All immigration and emigration events were also recorded. Individuals were classified as immigrants when they left their natal group and joined a new group for at least 30 days, and as emigrants when they vanished from their group and were subsequently observed in another (including groups outside the study population) [40]. Thirty days were chosen to be consistent with previous work in this species [40] and others [41]. This allowed recording of the origin (natal or immigrant) for many breeders. During each breeding season (October–April), the reproductive output of all groups was recorded, noting for each litter the number of pups that emerged from the burrow and that survived to 12 months of age.

## 3. Statistical analysis

We collated relevant information from the databases and performed all analyses using R v.4.2.2 [42]. We conducted parametric tests where data fitted the relevant assumptions of normality and homogeneity of variance; otherwise, we used non-parametric tests.

### (a) Breeder changes

To examine whether current group members or outsiders were more likely to fill natural vacancies arising from the death/dispersal of a breeder or to displace an existing breeder, we ran a chi-square test using all breeder changes. We then explored whether the same patterns were apparent for changes in both female and male breeders. To investigate whether new male and female breeders differed in their origin status (natal versus immigrant), we conducted two further chi-square tests. The first examined all breeder changes where the new breeder's residency status was known, and the second focused on just the subset where the breeder was replaced internally (i.e. whether the new breeder was a natal individual or had previously immigrated into the group).

### (b) Short-term and long-term consequences of a breeder change

**Figure 1.**
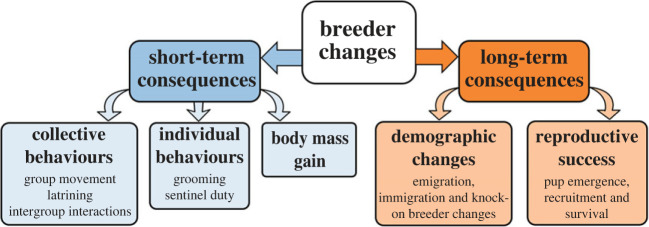
Schematic of the potential short-term and long-term responses to a breeder change that were analysed.

To determine the effect of breeder changes on short-term behaviours and body mass gain, and on long-term demographic changes and reproductive success (figure 1), we conducted linear and generalized linear mixed models (hereafter LMMs/GLMMs) using the package ‘lme4’ [43]. We fitted maximal models, including all fixed terms of interest and biologically relevant interactions (detailed below). Non-significant interactions were removed but, to avoid issues associated with stepwise model reduction, we did not simplify maximal models further [44,45]. For LMMs, the significance of main effects and interactions was evaluated using package ‘lmerTest’ [46], which conducts *F* tests using Satterthwaite's method for calculating denominator degrees of freedom [47]. For GLMMs, the significance of main effects and interactions was evaluated using package ‘car’ [48], which conducts Wald Chi-square tests using ANOVA. For GLMMs with a Poisson error structure, we checked for over-dispersion [49]. We explored significant interactions and three-level factors with Tukey's *post hoc* pairwise comparisons using the ‘emmeans’ package [50]. Where appropriate (for clusters of related response variables), significance levels were adjusted using a sequential Bonferroni procedure (see electronic supplementary material, tables S1 and S2).

To investigate short-term consequences, in terms of collective behaviours, individual behaviours and body mass gain, we analysed data from the fortnights before and after a breeder change (electronic supplementary material, table S1). We only included breeder-change events if groups were observed in both (matched) fortnightly periods. Groups were seen 4.5 ± 0.4 days (mean ± s.e.) in the fortnight before and 5.4 ± 0.5 days in the fortnight after a breeder change, with no significant difference between periods (Wilcoxon signed-rank test: *z* = 1.34, *n* = 33, *p* = 0.180). The number of breeder-change events differs for different analyses because of adjustments in the focus of data collection during the decade-long DMRP. To investigate long-term consequences, in terms of further demographic changes and reproductive success, we analysed data collected from groups experiencing a breeder change and from matched (control) groups with established dominant pairs (electronic supplementary material, table S2).

#### (i) Do breeder changes have short-term consequences for collective behaviour?

We compared group movement and territorial activity—latrining (scent-marking at communal sites) and IGIs—in the fortnight before and after a breeder change (electronic supplementary material, table S1a). For group movement, we conducted an LMM on log-transformed travel distances calculated from GPS tracks (*N* = 303 daily tracks, 24 breeder-change events, nine groups); we used the mean for each group in each fortnight period. For group territorial behaviour, we conducted one LMM on square-root-transformed latrining rate (*N* = 228 bouts, 30 breeder-change events, nine groups) and another LMM on square-root-transformed IGI rate (*N* = 15 interactions, 30 breeder-change events, nine groups). Rates were calculated as the number of interactions observed divided by the number of observation days in each two-week period. In all models, we included period (pre/post breeder change) and group size as fixed effects. To determine whether sex of the changing breeder influenced behavioural consequences, we included a two-way interaction between period and breeder sex. Breeder-change event nested in group identity was included as a random factor in all models.

#### (ii) Do breeder changes have short-term consequences for individual behaviour?

We compared individual levels of within-group grooming and sentinel activity in the fortnight before and after a breeder change, assessing both absolute rates (electronic supplementary material, table S1b) and the proportion of the group's behaviour performed by an individual (electronic supplementary material, table S1c). We considered the response of three categories of individual: ‘new breeders’ (individuals replacing an outgoing breeder; i.e. subordinate during the first fortnight and dominant during the second); ‘remaining breeders’ (breeding individuals retaining their position, thus dominant in both periods); and ‘helpers’ (individuals who were subordinate throughout the comparison period). We did not consider outgoing breeders as they were only present during the first two-week period. For grooming behaviour (*N* = 741 bouts, 94 individuals, 21 breeder-change events, eight groups), we conducted an LMM of log-transformed mean individual rate and a binomial GLMM for the proportion of group grooming performed; the latter used ‘cbind’ to compare the number of grooming bouts in which an individual participated with the number when it was not involved. For sentinel behaviour (*N* = 2882 scan samples, 1909 sentinel bouts, 102 individuals, 22 breeder-change events, nine groups), we conducted two binomial GLMMs with the proportion of sentinel bouts performed by each individual as the response variable. The first model bound the number of scan samples when an individual was a sentinel with the number when that individual was not a sentinel, investigating absolute sentinel rate. The second model bound the number of scan samples when an individual was a sentinel with the number when a different individual was a sentinel, investigating the proportion of group sentinel behaviour performed. In all models, period (pre/post breeder change), group size, individual sex and individual category type (new breeders, remaining breeders and helpers) were included as fixed effects. To determine whether different categories of individual adjusted their behaviour differently, we included an interaction between period and individual category, as well as that between period and sex of the changing breeder. In all models, individual identity and breeder-change event nested in group identity were included as random terms; date was included as an additional random term in sentinel GLMMs.

#### (iii) Do breeder changes have short-term consequences for individual body mass gain?

We investigated whether morning body mass gain of the three categories of individual was affected by comparing data from the fortnight before and after a breeder change (electronic supplementary material, table S1d). We conducted an LMM on square-root-transformed mean body mass changes; we only included individuals where body mass changes were available in both fortnightly periods (*N* = 354 body mass records, 17 breeder-change events, 36 individuals, eight groups). Period (pre/post breeder change), group size, individual sex and individual category type (new breeders, remaining breeders and helpers) were included as fixed effects, alongside the interactions between period and individual category and between period and sex of the changing breeder. Individual identity and breeder-change event nested in group identity were included as random terms.

#### (iv) Do breeder changes have long-term demographic consequences?

First, we examined whether a breeder change increased the likelihood of further demographic changes (another breeder change, an emigration or an immigration) in the following three months (electronic supplementary material, table S2a). We conducted a binomial GLMM (0 = no event occurred, 1 = event occurred) to compare the likelihood of a further demographic event occurring in groups after a breeder change with the likelihood of a demographic event occurring in the same three-month period in a matched control group where no recent breeder change had taken place (*N* = 57 matched pairs, 10 groups). For each breeder-change occurrence, we selected as a control group that from the same period that had an established dominant pair (a pair that had already bred together in a previous breeding season) and with the closest group size to the one that experienced the breeder change. We then conducted two binomial GLMMs considering each sex separately to investigate whether the reduced group stability occurred following both male (*N* = 29 matched pairs) and female (*N* = 28 matched pairs) breeder changes (electronic supplementary material, table S2b). We conducted three additional analyses considering each type of demographic event separately, to investigate whether the increase in demographic events following a breeder change was driven by an increase in immigrations, emigrations and/or further breeder changes. As immigration and emigration models suffered from quasi-complete separation, we used McNemar related-samples tests. For the analysis of further breeder changes, we used a binomial GLMM (electronic supplementary material, table S2b), with breeding pair type (new, established) and group size (adult group size at the start of the three-month period) as fixed effects, and with breeding pair identity nested in group identity and matched pair identity included as random terms.

#### (v) Do breeder changes have long-term consequences for reproductive success?

First, to investigate whether a breeder change influenced reproductive success, we used a GLMM with a Poisson error structure and a log link function to compare the number of pups surviving to 12 months from the first litter produced by new dominant pairs with that of matched established dominant pairs that produced a litter in the same month and year, thus controlling for temporal and spatial environmental variation (*N* = 30 matched pairs, 10 groups, seven breeding seasons; electronic supplementary material, table S2a). Established pairs were those that had already bred together in a previous season; we selected as the matched control group that with the closest group size to the one with a new pair. We then conducted a further two Poisson GLMMs (electronic supplementary material, table S2c) to investigate whether the change of a particular sex of breeder influenced the number of pups surviving to 12 months (new compared to established female breeders: *N* = 16; new compared to established male breeders: *N* = 14). Finally, we conducted two GLMMs (electronic supplementary material, table S2c) to examine whether the difference in the number of pups surviving for new compared to established female breeders resulted from a difference in the number emerging from the burrow (Poisson error structure and a log link function) and/or the post-emergence survival rate (binomial structure, using the ‘cbind’ function—i.e. proportion of emerging pups that survived to 12 months). In all models, we included pair type (new, established) and adult group size as fixed effects, with breeding pair identity nested in group identity and matched pair identity included as random terms. For the model considering number of emerging pups, we used adult group size at emergence; for models considering absolute or proportional pup survival across a 12-month period, we used weighted group size as a more accurate reflection of potential helper effort.

Finally, we considered possible reasons for a difference in reproductive success depending on the sex of the breeder that changes (electronic supplementary material, table S2d). To investigate whether new breeders were younger than established breeders, we used LMMs with square-root-transformed age in days (females: *N* = 16 pairs; males: *N* = 14 pairs). When exact age was unknown, it was calculated as the interval between the individual being encountered in the focal group (we assumed an age of 2 years for individuals first seen as adults) and the birth of the litter in question. To investigate whether new breeders were lighter than established breeders, we used LMMs on square-root-transformed body mass (females: *N* = 14 pairs; males: *N* = 9 pairs). For females, we used body mass data in the month before they were pregnant with the relevant litter; for males, we used body mass data in the month before the birth of the relevant litter.

### (c) Relative importance of breeder changes from outside the group

Since dispersal is costly, it is possible that any short-term and long-term consequences of breeder changes are driven by those occurrences when a breeder is replaced by an individual immigrating into the group at that point in time, rather than due to breeder changes *per se*. To assess this possibility, we repeated all analyses described above but excluding breeder-change events where the new breeder had just arrived from outside the group.

## 4. Results

### (a) Breeder changes

**Figure 2.**
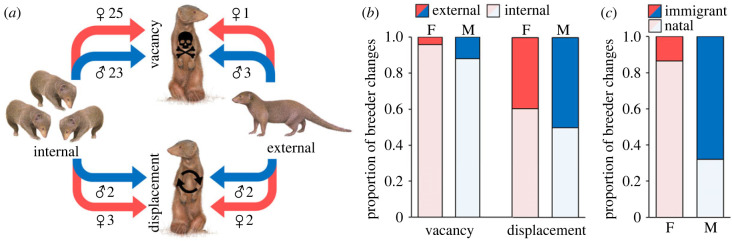
Routes to a breeding position in dwarf mongoose groups. (*a*) In both females (red) and males (blue), most changes in breeder (arrows) occurred after the death/dispersal of the existing breeder created a vacancy, though rivals sometimes actively displaced the incumbent. New breeders were mostly current group members (internal) rather than outsiders (external). *N* = 61 dominance changes, 11 groups. (*b*) Proportion of occasions when a new female (F) or male (M) breeder was an existing group member (internal) or an outsider (external), depending on whether there was a vacancy or active displacement. (*a,b*) Considers all observed breeder changes (*N* = 31 females, 30 males). (*c*) Proportion of occasions when the internal new female (F) or male (M) breeder was a previous immigrant or a natal group member. (*c*) Considers only internal breeder changes where the origin (natal versus immigrant) of the new breeder was known (*N* = 15 females, 16 males). Original mongoose artwork by Martin Aveling.

We observed 61 changes in the dominant breeding pair (31 females, 30 males) over 62 group-years in 11 groups (mean ± s.e. = 5.5 ± 0.9 changes per group, range = 0–11; electronic supplementary material, figure S1), with groups stable for 291 ± 53 days. Most breeder changes (85%) followed the creation of a vacancy due to the death or dispersal of an existing breeder; only relatively rarely (15% of changes) was an existing breeder actively challenged and displaced by another individual (figure 2*a*). Consequently, breeder changes tended to be quick, with the new breeder apparent within 24 h. Considering all breeder changes, the new breeder was frequently (87% of cases) an individual already in the group (internal); only 13% of new breeders were outsiders (external) (figure 2*a*). However, while breeding vacancies were more likely to be filled internally than externally, current group members and outsiders were similarly likely to displace an existing breeder (chi-square test: $\chi_{1}^{2}=9.09$, *p* = 0.002; figure 2*a*). This pattern was similar for both females and males (figure 2*b*), but statistically significant only for the former (females: $\chi_{1}^{2}=6.27$, *p* = 0.013; males: $\chi_{1}^{2}=3.69$, *p* = 0.055). Considering all breeder changes where the origin (natal versus immigrant) of the new breeder was known (*N* = 18 females, 21 males), there was a significant difference between the sexes ($\chi_{1}^{2}=11.30$, *p* = 0.0008): as expected given that dispersal is male-biased in this species [44], new breeding females were more likely to be natal group members (78%) than immigrants (22%), while the reverse was true for males (natal: 23%; immigrant: 77%). This sex difference remained when considering only individuals of known origin (*N* = 15 females, 16 males) that were already members of the group in which they became the breeder (i.e. internal replacements): new breeding females were more likely to have been born into that group (87%), whereas new breeding males were more likely to have been born into another group (69%) before immigrating into their current one at some point before they became the breeder ($\chi_{1}^{2}=9.76$, *p* = 0.002; figure 2*c*).

### (b) Do breeder changes have short-term consequences for collective behaviour?

**Figure 3.**
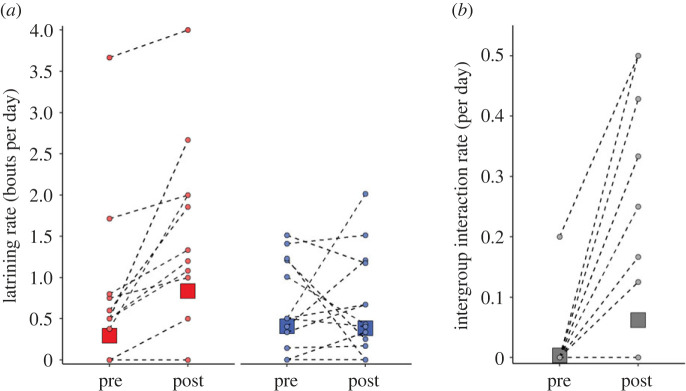
Collective behaviour consequences of a breeder change. (*a*) Group latrining rate (red: female breeder change; blue: male breeder change) and (*b*) intergroup interaction rate (all breeder changes) in the fortnight before (pre) and after (post) a breeder change; rates are given per day. Square points are back-transformed predicted means (± s.e.) from the mixed models presented in the electronic supplementary material, table S3; s.e. can fall within the bounds of the square point. Circular points are raw data for each breeder-change event (*N* = 30), with values from the same breeder-change event connected by dotted lines; in some instances, more than one breeder-change event has the same value, hence the number of dashed lines appears less than the sample size for matched periods.

In the fortnight after a breeder change, daily distance travelled by groups did not differ significantly from that in the fortnight before (LMM, period: *F* = 0.004, *p* = 0.950; period × breeder sex interaction: *F* = 2.260, *p* = 0.147; electronic supplementary material, table S3a). However, group territorial behaviour increased following a breeder change. Following a change in the female breeder, groups latrined at more than twice the rate of the fortnight before, but latrining rate did not differ significantly following a male-breeder change (period × breeder sex interaction: *F* = 7.472, *p* = 0.011; electronic supplementary material, table S3b; figure 3*a*; *post hoc* Tukey's (PHT) comparing periods, female-breeder change: *p* = 0.002; male-breeder change: *p* = 0.861). Moreover, there was a significant increase in the rate of intergroup interactions in the fortnight after a breeder change compared to the fortnight before (period: *F* = 6.012, *p* = 0.023; figure 3*b*), regardless of the sex of breeder that changed (period × breeder sex interaction: *F* = 0.018, *p* = 0.895; electronic supplementary material, table S3c).

### (c) Do breeder changes have short-term consequences for individual behaviour?

**Figure 4.**
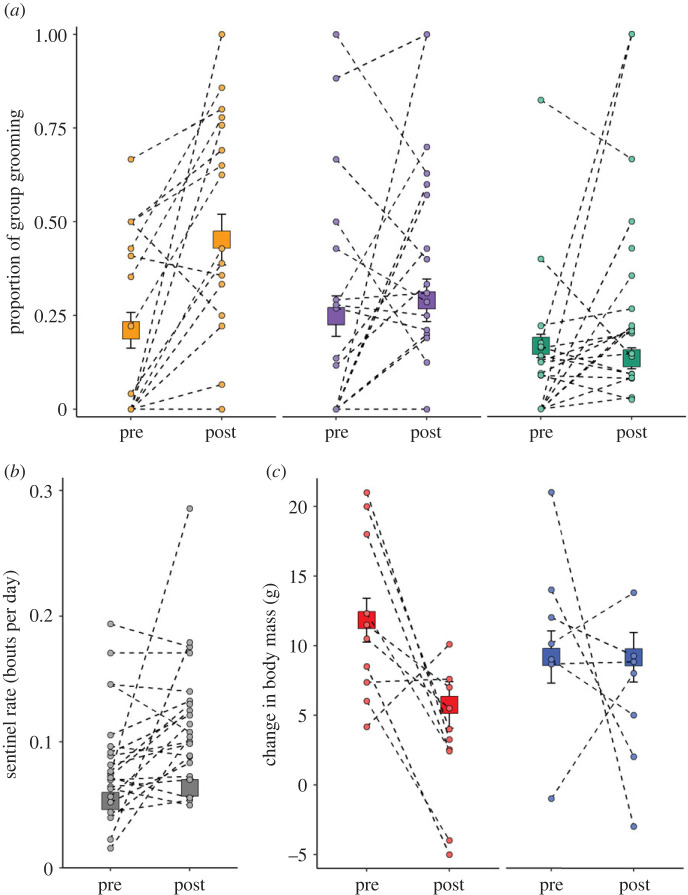
Individual-level consequences of a breeder change. (*a*) Proportion of group grooming (new breeders: orange; remaining breeders: purple; subordinate helpers: green), (*b*) individual sentinel rate (bouts per day) in the fortnight before (pre) and after (post) all breeder changes and (*c*) individual body mass change following a change in female (red) and male (blue) breeders. Square points are predicted means (± s.e.) from the mixed models presented in the electronic supplementary material, tables S4–S6; s.e. can fall within the bounds of the square point. Circular points are raw data for each breeder-change event, representing (*a*) the mean value for each category of individual in each breeder-change event and (*b,c*) the mean value across all categories of individuals for each breeder-change event. Dotted lines connect values from the same breeder-change event; in some instances, more than one breeder-change event has the same value, hence the number of dashed lines appears less than the sample size for matched periods. (*a*) *N* = 21 breeder-change events, 18 new breeders, 18 remaining breeders, 96 helpers; (*b*) *N* = 22 breeder-change events; and (*c*) *N* = 17 breeder-change events.

Individuals groomed at approximately 3.5 times the rate in the fortnight after a breeder change compared to the fortnight before (LMM, period: *F* = 20.888, *p* < 0.0001), irrespective of the sex of the breeder that changed (period × breeder sex interaction: *F* = 0.151, *p* = 0.698; electronic supplementary material, table S4a). While there was an overall difference between the three categories of individual (*F* = 6.905, *p* = 0.001; electronic supplementary material, table S4a)—remaining and incoming breeders groomed at twice the rate of helpers (electronic supplementary material, table S7a)—this difference was consistent before and after a breeder change (period × individual category interaction: *F* = 3.404, *p* = 0.035, not significant after Bonferroni correction; electronic supplementary material, table S4a). Considering proportional contributions to group grooming, there was a difference between the three categories of individuals in how their behaviour changed (GLMM, period × individual category interaction: *χ*^2^ = 30.144, *p* < 0.0001; electronic supplementary material, table S5a): new breeders performed twice the proportion of group grooming after newly acquiring breeding status (PHT: *p* < 0.0001), while the proportions contributed by remaining breeders (*p* = 0.374) and helpers (*p* = 0.114) were not significantly different from those in the fortnight before (electronic supplementary material, table S7b; figure 4*a*). Following a breeder change, all individuals increased their rate of sentinel behaviour by approximately 20% (GLMM, period: *χ*^2^ = 4.228, *p* = 0.040; figure 4*b*), with no significant difference in the increase between new breeders, remaining breeders and helpers (period × individual category interaction: *χ*^2^ = 4.619, *p* = 0.099) or depending on the sex of the breeder that changed (period × breeder sex interaction: *χ*^2^ = 0.330, *p* = 0.566; electronic supplementary material, table S4b). While there was an overall difference between the three categories of individuals in their proportional contributions to sentinel activity (*χ*^2^ = 38.914, *p* < 0.0001; electronic supplementary material, table S5b)—remaining breeders performed twice as much group sentinel behaviour as new breeders and helpers (electronic supplementary material, table S7d)—this did not change significantly from before to after a breeder change (period × individual category interaction: *χ*^2^ = 5.513, *p* = 0.064; electronic supplementary material, table S5b).

### (d) Do breeder changes have short-term consequences for individual body mass gain?

Body mass gain was affected by the interaction between period and breeder sex (LMM: *F* = 4.034, *p* = 0.048; electronic supplementary material, table S6; figure 4*c*): individuals gained approximately 6 g less mass per morning foraging session following a change in the female breeder (PHT comparing periods: *p* = 0.006) but not the male breeder (*p* = 0.967; electronic supplementary material, table S7e). There was no significant difference in this reduction in body mass gain between new breeders, remaining breeders and helpers (period × individual category interaction: *F* = 0.265, *p* = 0.768; electronic supplementary material, table S6).

### (e) Do breeder changes have long-term demographic consequences?

**Figure 5.**
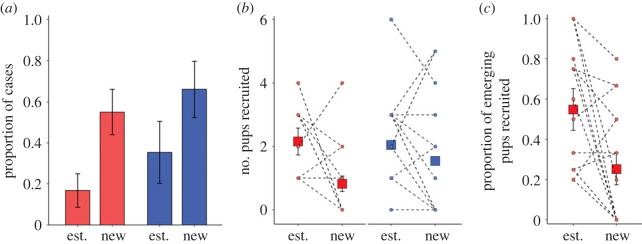
Demographic and reproductive consequences of a breeder change. (*a*) Likelihood of a demographic change within three months of a female-breeder change (red, *N* = 28 matched pairs) or a male-breeder change (blue, *N* = 29 matched pairs) compared to when there has been an established (est.) breeding pair. Shown are predicted means (± s.e.) from the mixed models presented in the electronic supplementary material, table S8. (*b*) Pup recruitment (number surviving to 12 months of age, back-transformed) of new (first breeding attempt) and established (bred in previous breeding season) female breeders (red, *N* = 16 matched pairs) and male breeders (blue, *N* = 14 matched pairs), and (*c*) post-emergence pup survival for new compared to established female breeders (*N* = 16 matched pairs). In (*b*,*c*), square points are predicted means (± s.e.) from the mixed models presented in the electronic supplementary material, table S9; s.e. can fall within the bounds of the square point. Circular points are raw data for each breeder-change event, with values from the same breeder-change event connected by dotted lines; in some instances, more than one breeder-change event has the same value, hence the number of dashed lines appears less than the sample size for matched periods.

In the three months after a breeder change, groups were more likely than control groups to experience another demographic change (GLMM: *χ*^2^ = 6.527, *p* = 0.011; electronic supplementary material, table S8a). But, whereas there was a threefold increase in the likelihood of further demographic change following a female-breeder change (*χ*^2^ = 5.910, *p* = 0.015; electronic supplementary material, table S8b; figure 5*a*), there was no significant effect following a male-breeder change (*χ*^2^ = 2.037, *p* = 0.153; electronic supplementary material, table S8c; figure 5*a*). The overall result was not due to any significant difference in the likelihood of immigration (McNemar related-samples test: *χ*^2^ = 2.769, d.f. = 1, *p* = 0.096) or emigration (*χ*^2^ = 0.071, d.f. = 1, *p* = 0.789). Rather, it was because a breeder change triggered a domino effect in terms of further breeder changes, with another change within three months five times more likely than in control groups (GLMM: *χ*^2^ = 8.721, *p* = 0.003; electronic supplementary material, table S8d).

### (f) Do breeder changes have long-term consequences for reproductive success?

Groups with new dominant pairs raised one less pup to 12 months than those with established pairs (GLMM: *χ*^2^ = 6.931, *p* = 0.008; electronic supplementary material, table S9a). The negative effect on breeding success was only apparent following a change in the female breeder (*χ*^2^ = 9.108, *p* = 0.003; electronic supplementary material, table S9b; figure 5*b*); pup recruitment did not differ significantly between groups with new compared to established male breeders (*χ*^2^ = 0.935, *p* = 0.070; electronic supplementary material, table S9c; figure 5*b*). The negative effect on pup recruitment following a female-breeder change was not the result of significantly fewer pups emerging from the burrow (*χ*^2^ = 2.120, *p* = 0.138; electronic supplementary material, table S9d), but because of a lower post-emergence survival rate to 12 months of age (25%) compared to that in control groups (54%; *χ*^2^ = 5.999, *p* = 0.014; electronic supplementary material, table S9e; figure 5*c*).

Considering possible reasons why a change in female breeder affected reproductive success, we found that first-time female breeders were younger (mean ± s.e. = 870 ± 72 days) than established female breeders (1187 ± 42 days; LMM: *F* = 15.177, *p* = 0.002; electronic supplementary material, table S10a), but did not differ significantly in body mass (*F* = 3.033, *p* = 0.115; electronic supplementary material, table S10b). By contrast, there was no significant difference in the age of first-time (1364 ± 177 days) and established (1598 ± 189 days) male breeders (*F* = 2.359, *p* = 0.150; electronic supplementary material, table S10c), and new male breeders were actually heavier than their more established counterparts (*F* = 10.396, *p* = 0.006; electronic supplementary material, table S10d).

### (g) Relative importance of breeder changes from outside the group

For the majority of short-term and long-term consequences, we found qualitatively the same results as above when using the datasets without those breeder-change events where an outsider had immigrated into the group at that point. For collective behaviours, the same factors as above were significant when considering the change in group movement (electronic supplementary material, table S11a), latrining (electronic supplementary material, table S11b) and IGIs (electronic supplementary material, table S11c). Likewise, we also found qualitatively the same results as above for individual grooming rate (electronic supplementary material, table S12a) and the proportion of group grooming (electronic supplementary material, table S13a). Using the full dataset (above), we found that all three categories of individual increased their sentinel rate following a breeder change but, having removed immigrant new breeders from the dataset, it was remaining breeders rather than new breeders or helpers who increased their rate and proportional sentinel contributions after a breeder change (electronic supplementary material, tables S12b, S13b and S15b–c). With all breeder changes included (above), individuals gained less body mass after a female-breeder change, whereas with the reduced dataset this effect was apparent after changes in breeders of both sexes (electronic supplementary material, table S14). Qualitatively the same results as above were found when considering the likelihood of further demographic changes (electronic supplementary material, table S16) and reproductive success (electronic supplementary material, table S17).

## 5. Discussion

Using long-term data from a wild population of cooperatively breeding dwarf mongooses, we found that a change in one of the group's dominant breeding pair led to alterations in both collective and individual behaviour, as well as reduced body mass gain, further demographic changes and decreased reproductive success. The documented effects of social instability were particularly apparent when it was the female breeder who changed; the effects were generally apparent and not just driven by those (relatively rare) occurrences when an individual took over the breeding position from outside the focal group. While there is a growing literature considering the consequences of social instability [6,7], cooperatively breeding species have rarely been considered. This is surprising given that social stability is considered a key factor in the evolution of cooperative societies and is a potentially important contributor to fitness in such species [22,23].

Past work on plural or pair-breeding species has reported a reduction in cooperative behaviours following periods of social instability [12,13,51]. By contrast, we found an increase in cooperation: dwarf mongoose group members engaged in more IGIs and exhibited elevated levels of grooming and sentinel activity in the fortnight following a change in either breeder compared to the fortnight before; they also increased their collective latrining behaviour following a female-breeder change. Further work would be needed to determine how long these changes last. As scent-marking can facilitate social bonding [52], increased latrining may reflect an attempt to restore group cohesion following social disruption. Alternatively, more latrining and IGIs may result from a greater need for territorial defence, since breeder-change events were generally associated with a reduction in group size and relative group sizes can influence intergroup conflict levels [53,54]. Groups that have had a recent breeder change might therefore have proactively increased defensive effort to discourage intrusions or been forced to counter an escalation in territorial behaviour by neighbours during unstable periods. Increased grooming following a breeder change may serve to enhance social cohesion and stabilize disturbed social networks [55,56], as well as alleviate heightened stress levels associated with social instability [8,10,57]. The increase in sentinel rate was likely due both to an overall reduction in group size and because the lost individual is one that contributed disproportionately to that behaviour; dominant dwarf mongooses perform almost twice as many bouts as subordinates [28,38]. Greater vigilance could also represent greater perceived predation risk following groupmate loss [56] or an increased need to gather information about rival-group activity or own groupmates [39,58]. Several studies of cooperatively breeding species have reported an increase in inter- and intra-sexual conflict in the wake of breeder loss, as remaining subordinates compete for access to the breeding vacancy [15,16,32]. But to the best of our knowledge, no work has previously investigated how cooperative behaviours change once a breeding vacancy in such societies has been filled by a new individual.

There was some intragroup variation in the behavioural adjustments seen following a breeder change, especially with respect to grooming: while new breeders contributed a greater proportion of group grooming once they had obtained their new status, there was no significant change in the proportional contributions of remaining breeders and helpers. As the category of individual that has experienced a personal change in dominance-hierarchy position, incoming breeders may invest more in grooming to reinforce their new position, establish a strong bond with their partner [59] and deter subordinate contests [60,61], to incentivize subordinate helpers during unstable periods [62] or to reward them for their cooperative contributions [34,39]. Explaining variation in cooperative contributions has been a key focus in evolutionary biology, with a range of other individual, social and environmental factors identified as important [63–66]. Our work on dwarf mongooses demonstrates that demographic changes can also alter the expression of cooperative behaviours with, for example, recent immigrants conducting lower-than-expected levels of sentinel behaviour [40] but all group members increasing this behaviour following a breeder change (this study).

Beyond behavioural alterations, we found negative effects on body mass gain, group stability and reproductive success following a change in the female, but not the male, breeder. There is a similar sex effect in grey wolves, where mortality of the female breeder was more likely than loss of the male breeder to result in pack dissolution [21], although there were no differences in wolf pup survival depending on the sex of the lost breeder [67]. The lower body mass gains that we documented in dwarf mongooses following a breeder change likely resulted from a loss of foraging time due to increases in territory defence, grooming and sentinel behaviour; increased stress levels may also play a role [68]. The generation of more instability—an initial breeder change triggered a greater likelihood of further such changes—contrasts recent studies that emphasized the robustness of groups to demographic changes when considering only social networks [55,56,69]. In dwarf mongoose groups, most replacement breeders are existing group members, but new female breeders are more likely to have been born into that group while new male breeders are more likely to have arrived from elsewhere. In general, greater social disruption might be expected following the loss of a philopatric breeder, as longer-term residents may be able to foster stronger relationships and so be more central to group networks [70,71]. Moreover, there may be greater rank uncertainty among females, such that it is less clear-cut that the new breeder really is the most dominant remaining individual; ongoing contests between the new female breeder and same-sex group members (e.g. sisters) might cause further disruption.

Prolonged social instability could be one reason for the lower reproductive success of groups with a new female breeder. In principle, lower post-emergence pup survival might arise if helpers in unstable groups provided less alloparental care [14], either because of increased time spent performing other cooperative acts (territory defence, grooming and sentinel behaviour) or due to body mass losses that require compensatory foraging [71,72]. The reproductive effect might also be the consequence of new female breeders being younger than more established female breeders, which was not the case for new male breeders. Younger females are likely less experienced at caring for young, have less familiarity with their mate and have less knowledge of the territory, all of which might influence offspring survival [73,74]. First-time breeders in other species [75,76], including cooperative breeders [77], have lower reproductive success, although those findings were not necessarily limited to females. In dwarf mongoose groups, older, higher ranking subordinate females may sometimes produce litters in synchrony with the dominant female [78]. In principle, the lower pup recruitment that we document in groups with new breeding pairs could stem in part from a reduction in subordinate female births, although we would then expect to see fewer pups initially emerging from breeding burrows, which was not the case. Ultimately, genetic data are needed—preliminary analyses from our study population suggest that at least 90% of pups are produced by the dominant pair (unpublished data), but we do not have DNA from the study animals throughout the study period—if we are to determine the cause of the reproductive success effects that we find.

In conclusion, our study demonstrates that a breeder change in wild dwarf mongoose groups, especially that of the female member of the dominant pair, can have a range of behavioural, body mass, demographic and reproductive consequences across different timeframes. Studies focusing just on short-term effects and/or a single type of behavioural change might not reveal these varied costs of social instability and may even give the impression of structural resilience [56,69]. Our findings of both short- and long-term consequences of breeder changes support the idea that stability and cooperation are strongly linked [79] and provide potential reasons for previously documented health and fitness benefits of social stability [1–3]. Future work could consider consequences for group members of different dominance status, age and experience, as well as changes arising from other forms of social instability (e.g. loss of non-breeding group members, changes in dominance hierarchies). Demographic changes are an inherent element of all social systems and understanding the impacts of instability will enhance our ability to predict the consequences. These consequences may be particularly important in species with skewed reproduction or complex social structures, where the loss of key individuals may disproportionately affect those remaining and, ultimately, group survival and population dynamics [21].

## Data Availability

Data are available from the Dryad Digital Repository: https://doi.org/10.5061/dryad.7h44j1005 [80]. Additional information is provided in the electronic supplementary material [81].
